# Ethoxy Acetalated Dextran-Based Biomaterials for Therapeutic Applications

**DOI:** 10.3390/polym16192756

**Published:** 2024-09-29

**Authors:** Branden Joshua Damus, Nzube Ruth Amaeze, Eunsoo Yoo, Gagandeep Kaur

**Affiliations:** 1 Department of Microbiology and Immunology, University of Miami, Coral Gables, FL 33146, USA; 2 Department of Chemistry, College of Arts and Sciences, Howard University, Washington, DC 20059, USA; 3 Department of Chemical, Biological, & Bioengineering, North Caroline Agricultural and Technical State University, Greensboro, NC 27411, USA

**Keywords:** ethoxy acetalated dextran, biomaterials, drug delivery, immunotherapy, tissue engineering, biomedical applications

## Abstract

A novel class of pH-responsive polymers, acetalated dextran, has emerged in the field of biomaterials. These versatile materials are derived from dextran through a simple acetalation reaction, allowing for the creation of polymers with a tunable release profile which allows the controlled release of encapsulated therapeutics in response to acidic environments. Despite their recent introduction, acetalated dextran has rapidly garnered significant interest due to its potential for various therapeutic applications. This review delves specifically into the recent advancements of ethoxy acetalated dextran or Ace-DEX, a particular acetalated dextran with a distinct advantage: its degradation products (acetone and ethanol) are less toxic compared to other variants that produce methanol. The focus of this review is the diverse range of biomedical applications currently being explored for Ace-DEX-based scaffolds. Finally, this review concludes by addressing the existing challenges associated with Ace-DEX and outlining potential future research directions within this promising field.

## 1. Introduction

**Figure 1:**
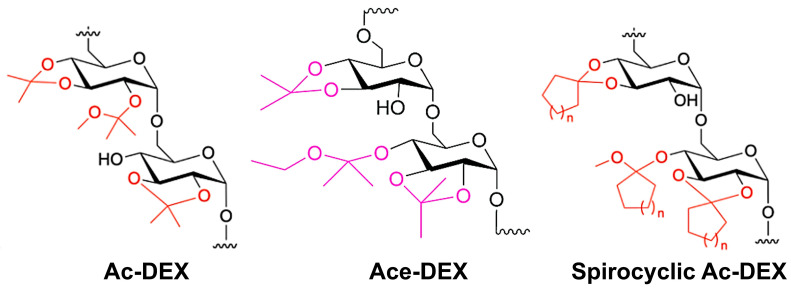
Chemical structures of acetalated dextran derivates: methoxy (Ac-DEX), ethoxy (Ace-DEX), and spriocyclic acetalated dextran.

Acetalated dextran-based biomaterials have emerged as an appealing area of research in the field of therapeutic applications. Due to its hydrophobic nature, acetalated dextran stands out as one of the most widely investigated biomaterials among several other dextran derivatives (Figure 1). Acetalated dextran was synthesized through a straightforward one-step reaction involving dextran, 2-methoxypropene, and a pyridinium p-toluenesulfonate (PPTS) catalyst and first reported by Bachelder et al. [1]. This reaction leads to the acetalation of hydroxyl groups on dextran chains. Interestingly, the resulting methoxy acetalated dextran (Ac-DEX) polymer becomes insoluble in water, yet is readily soluble in organic solvents like ethyl acetate and dichloromethane. A critical property of Ac-DEX lies in its acid-triggered degradation. Under acidic conditions, Ac-DEX hydrolyzes back to dextran, releasing methanol and acetone as byproducts [1]. This unique combination of properties—facile synthesis, hydrophobicity, solubility in organic solvent, and acid-triggered degradation—presents Ac-DEX as a highly attractive biomaterial for drug loading [1,2]. By employing precipitation or emulsion techniques, drugs can be encapsulated within the Ac-DEX scaffolds. The acid-sensitive nature of the acetal groups allows for controlled drug release in specific acidic environments, such as inflamed areas, the tumor microenvironment, and/or endosomal compartments [3]. A key structural feature of Ac-DEX-based biomaterial is the ratio of acyclic and cyclic acetals, which has the ability to control the polymer degradation rate from hours to days [4]. Acyclic acetals decay rapidly into acetone and methanol, while cyclic acetals undergo a slower hydrolysis process, releasing only acetone [4]. By carefully controlling the ratio of these acetal types, a precisely tailored polymer degradation half-life can be achieved, which offers a powerful tool for optimizing drug delivery. However, the presence of methanol presents a challenge for in vitro and in vivo studies due to its cytotoxicity.

**Figure 2:**
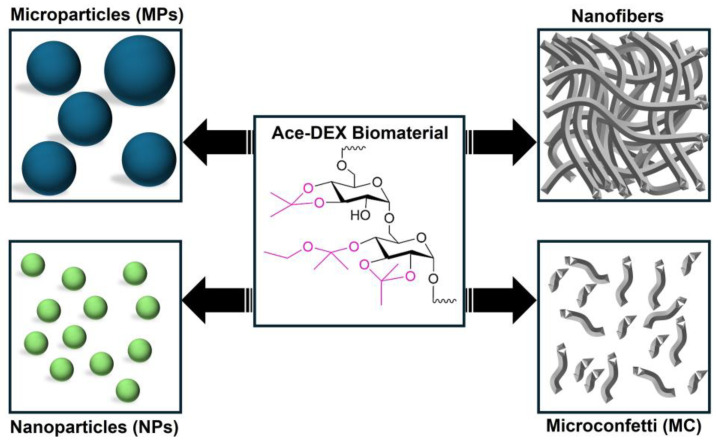
Ace-DEX can be fabricated into nanoparticles (NPs), microparticles (MPs), nanofibers, and microconfetti (MC) using various fabrication methods.

To tackle this issue, ethoxy acetalated dextran (Ace-DEX) was synthesized using 2-ethoxypropene by Kauffman et al. [5]. This derivative boasts similar physicochemical properties and exhibits comparable pH-dependent degradation along with low to no cytotoxicity [5]. The key difference lies in the degradation metabolites: Ace-DEX breaks down into acetone and ethanol, instead of the methanol and acetone produced by Ac-DEX [5]. Ethanol exhibits significantly lower cytotoxicity compared to methanol. This is an advantage, particularly when considering high-dose administration or extended treatment durations often required for chronic conditions as compared to Ac-DEX and traditional polymers like PLGA, which generate acidic microenvironments upon degradation [6,7,8]. Ace-DEX can be easily fabricated into nanoparticles, microparticles, nanofibers, and microconfetti (Figure 2) [2,9]. The detailed synthesis and scaffold fabrication methods for the acetalated dextran (methoxy (Ac-DEX), ethoxy (Ace-DEX), and spriocyclic acetalated dextran) has been previously published elsewhere in the literature [9].

Ace-DEX’s unique properties, such as facile synthesis, controlled degradation profile, acid sensitivity, and easy fabrication, allow it to be fashioned into scaffolds with precisely controlled characteristics, which results in a promising broad range of therapeutic applications. These scaffolds also have the ability to mimic the natural extracellular matrix (ECM), establishing a nurturing and favorable environment for cellular growth, differentiation, and successful integration within surrounding tissues [10]. This capability makes these scaffolds prime candidates for regenerative medicine and tissue engineering applications. This review presents the latest cutting-edge advancements in Ace-DEX biomaterials for a diverse range of therapeutic applications. These include drug delivery, vaccines, cancer immunotherapy, and various other areas where Ace-DEX’s versatility holds immense promise (Table 1).

## 2. Drug Delivery

**Figure 3:**
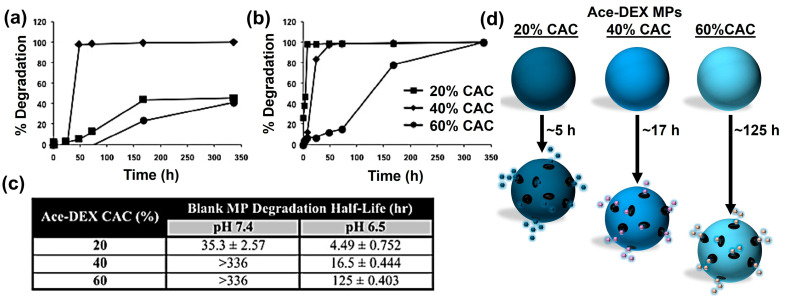
Degradation rate of blank Ace-DEX MPs with variable relative cyclic acetal coverage (CAC) at (**a**) pH 7.4 and (**b**) pH 6.5; (**c**) table showing degradation half-life for different CAC Ace-DEX MPs; (**d**) a schematic representation of Ace-DEX MP degradation. Reproduced and modified image from ref. [30] with permission from Elsevier, Copyright © 2018.

Ace-DEX has become a prominent choice for drug delivery platforms for various therapeutic applications, ranging from the treatment of infectious diseases to cancer treatment, due to the controllable degradation rate [11,12,29]. Ace-DEX nanoparticles (NPs) and microparticles (MPs) display considerable promise as carriers for drug delivery due to their distinctive combination of properties including a typical size range spanning from 140 to 350 nm for NPs, 0.5 to 2 µm for MPs, biodegradability, controlled degradation, and acid sensitivity [5,9]. Specifically, the acid sensitivity enables tunable degradation kinetics, allowing for specific control over the rate of degradability. Furthermore, Ace-DEX NP and MP degradation rates can be specifically modulated by adjusting the proportion of acyclic and cyclic acetals within the biomaterial structure. This strategic manipulation allows for fine-tuning of the pH at which biomaterial degradation rapidly occurs, providing a deep understanding for controlled drug release. Chen et al. investigated the degradation behavior of Ace-DEX in a recent study where four distinct molecular weights, 10, 71, 500, and 2000 kDa with 20%, 40%, and 60% of cyclic acetals were examined (Figure 3) [30]. At pH 5, all combinations of 20% cyclic acetals exhibited fast degradation with a half-life of approximately 0.25 h. For 40% cyclic acetals, the degradation rate was found to be inversely related to the polymer molecular weight at pH 5. For 60% cyclic acetals, the degradation rate was similar for all molecular weights of polymers with 21 h as a half-life.

**Figure 4:**
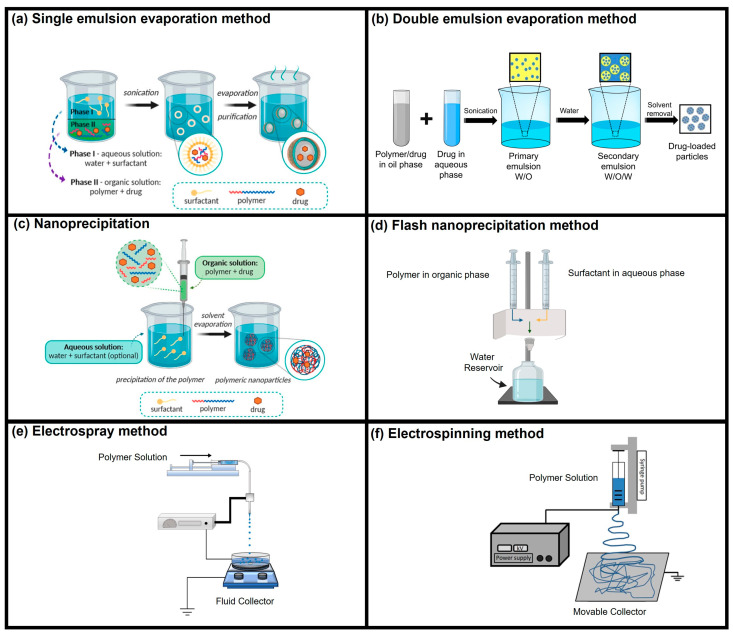
Reproduced and modified from ref. [32], published by Springer Nature, under the Creative Commons Attribution-CC by 4.0 Attribution 4.0 International Deed. Fabrication methods used for drug delivery applications using Ace-DEX biomaterial. Reproduced from ref. [33], published by MDPI, under the Creative Commons Attribution-CC by 4.0 Attribution 4.0 International Deed. Reproduced and modified image from ref. [34] with permission from Elsevier, Copyright © 2022. Reproduced and modified image from ref. [35] with permission from Elsevier, Copyright © 2021. Reproduced and modified image from ref. [36] with permission from Elsevier, Copyright © 2015.

Ace-DEX NPs and MPs exhibit several compelling advantages when used as drug delivery vehicles. Ace-DEX NPs and MPs take advantage of their acid sensitivity to achieve controlled and site-specific drug release. Within endosomes, where the pH is approximately 5.0 (acidic), Ace-DEX NPs and MPs quickly release their cargo. On the other hand, at physiological pH levels, the release rate is significantly slower [30]. This pH-dependent behavior ensures specific delivery of drugs or therapeutic agents to targeted sites. Besides controlled release, Ace-DEX NPs and MPs offer rational advantages related to cargo stability and safety. Ace-DEX NPs and MPs support the stability of encapsulated cargo for extended storage periods. Their inherent properties lower the reliance on cold-chain storage, simplifying logistics and distribution. The Ace-DEX NP and MP formulation also allows for terminal sterilization [31]. This crucial step makes sure that the resulting therapeutic product is safe and effective. Ace-DEX NPs and MPs are versatile carriers for drug delivery, and several fabrication methods including both traditional and innovative techniques like nanoprecipitation, spray drying, electrospray, and single and double emulsion can be employed to load drugs or therapeutic payloads into them (Figure 4) [9]. It is notable to acknowledge that commonly used fabrication techniques like single and double emulsion methods rely on organic solvents which are toxic, such as chloroform or dichloromethane. Moreover, these methods involve multi-step processing to achieve a stable drug or payload containing NPs and MPs. Recently, Hughes et al. established a straightforward and efficient approach called flash nanoprecipitation (FNP) for loading therapeutic payloads in Ac-DEX NPs [32]. FNP utilizes a confined impinging jet mixer, which ensures precise and efficient mixing of the components. The controlled environment within the mixer contributes to uniform nanoparticle formation. Unlike traditional methods that often rely on potentially toxic organic solvents, FNP utilizes ethanol to encapsulate various payload types—hydrophobic compounds, hydrophilic molecules, and biomacromolecules. Ac-DEX NPs prepared using the FNP method could be lyophilized and stay stable for a year with more than 80% load retention. FNP could certainly be employed for payload encapsulation in Ace-DEX NPs and MPs.

### 2.1. Infectious Disease Treatment

Size-based passive targeting using acid-sensitive Ace-DEX MPs has been explored for many infectious diseases. Hoang et al. demonstrated that AR-12-loaded Ace-DEX MPs significantly mitigated AR-12 cytotoxicity while preserving its capacity to increase Salmonella clearance in human monocyte-derived macrophages (hMDMs) through autophagy [13]. AR-12 (OSU-03012) has anticancer, anti-microbial, and anti-viral properties. It is known for modulating host–cell activities by inhibiting Akt pathway activation [37]. While AR-12 has demonstrated efficacy in enhancing various intracellular pathogen clearance, the hydrophobic nature and inherent toxicity of this molecule imposes limitations on its in vivo therapeutic effectiveness. Encapsulating AR-12 in Ace-DEX MPs mitigates these issues and effectivity utilizes its anti-microbial qualities against Salmonella infection [13]. Apart from Salmonella infection, AR-12-Ace-DEX MPs were also explored in controlling Francisella infection in hMDMs and in a tularemia mouse model [14]. It was reported that Ace-DEX MPs exhibited passive targeting towards phagocytes, because other cell types were incapable of engulfing particles of equivalent size. It was noticed that the co-delivery of AR-12 alongside a suboptimal dose of gentamicin resulted in improved protective effects in the tularemia mouse model as compared to either drug administered alone. AR-12-Ace-DEX MPs has also been explored as a potential treatment for visceral leishmaniasis, a protozoan parasitic disease, transmitted by female phlebotomine sandfly bites when infected [12]. Beyond its use with AR-12, Ace-DEX has demonstrated its versatility by enhancing the efficacy and reducing the toxicity of other host-mediated compounds upon encapsulation and delivery [29].

### 2.2. Cancer Chemotherapy

Collagenase-loaded Ace-DEX NPs were explored to tackle the tumor tissue’s dense extracellular matrix (ECM) [15]. Collagenase is a protein which acts as an enzyme in the presence of Zn^2+^ ions to break down collagen in the body. It is a pH- and temperature-sensitive protein. It is important to note that tumor tissues generally have extremely dense ECM made up of collagen which protects it and makes it difficult to treat. Collagenase-loaded Ace-DEX NPs when utilized in combination with doxorubicin (DOX)-loaded liposome (DOX-Lipo) were able to penetrate more efficiently in tumor tissue and reduce the tumor by 2.8-fold without any toxicity [15]. The ability of Ace-DEX MPs to encapsulate sensitive protein molecules, maintain their bioactivity, and deliver the payload in the acidic microenvironment of a tumor has been explored in this study.

Ace-DEX-based scaffolds like microconfetti and nanofibers have also been utilized for anticancer drug encapsulation and delivery as a cancer chemotherapy in many reports in the literature [11,16,17]. Graham-Gurysh et al. exploited acid-sensitive paclitaxel-loaded Ace-DEX electrospun nanofibers as a probable glioblastoma (GBM) interstitial therapy. GBM is an aggressive brain tumor characterized by an exceptional and challenging acidic microenvironment [16]. These nanofibers were engineered to degrade and release the encapsulated paclitaxel (PTX, a potent anticancer drug) in response to the acidic microenvironment of a GBM tumor. This targeted release approach has the capacity to enhance drug delivery specifically to the acidic tumor site, reducing exposure to healthy tissues and potentially decreasing systemic side effects. Similarly, doxorubicin-loaded Ace-DEX electrospun-nanofiber-based implants have also been explored as a potential platform for GBM interstitial therapy [11].

Ace-DEX has emerged as a promising biomaterial for drug delivery applications due to its unique biodegradability profile. Unlike many polymers with pre-defined degradation times, Ace-DEX offers a high degree of tunability. By modifying its chemical composition, Ace-DEX can be engineered to degrade at various rates, ranging from hours to days to months [30]. This tailored degradation characteristic transforms Ace-DEX into a versatile delivery platform. It allows specific control over the release rate of encapsulated drugs, ensuring optimal therapeutic effects. This feature becomes particularly essential in the context of combination therapies. By precisely controlling the release rates of different drugs within the Ace-DEX carrier, the drug-to-drug ratio can be optimized, potentially leading to enhanced efficacy and reduced side effects. Graham-Gurysh et al. utilized the electrospinning fabrication method to engineer PTX- and everolimus (EVR)-encapsulated Ace-DEX nanofibers with similar release profiles (approximately 3% per day) [17]. The treatment with a combination of Ace-DEX-PTX and Ace-DEX-EVR scaffolds widely reduced tumor recurrence and enhanced progression-free survival in mouse models mimicking GBM. These findings emphasize the potential advantages of combination therapy delivered interstitially (directly into the tumor site) and demonstrate the utility of Ace-DEX as a tunable platform for specifically coordinated drug delivery in GBM treatment.

### 2.3. Inflammatory Disease Treatment

Most inflammatory diseases have no cure, but treatment can help with the symptom management. A key advantage of Ace-DEX biomaterial compared to some other polymeric scaffolds lies in its degradation products. Unlike the acidic byproducts generated during the degradation of poly(lactic-co-glycolic acid) (PLGA), a commonly used polymer, Ace-DEX degrades into neutral or slightly basic compounds [6,7,8]. This is very significant as an acidic microenvironment produced by polyester can be detrimental to surrounding tissues, may hinder cell viability, and elevate inflammation. Conversely, the neutral to slightly basic degradation products of Ace-DEX promote better biocompatibility and potentially support cellular activity within the implantation site. Anti-inflammatory molecules (such as BRP-187 and BRP-201) have been encapsulated into Ace-DEX scaffolds and explored as potentially better inflammatory disease treatment options as compared to currently used biomaterials [18,19]. A comparative study reported by Behnke et al. demonstrated that Ace-DEX NPs were better than PLGA NPs with respect to drug (BRP-187, a highly lipophilic and anti-inflammatory molecule) loading when different methods such as emulsion solvent evaporation, microfluidics, and nanoprecipitation were employed [18]. It was also noted that Ace-DEX NPs loaded with BRP-187 did not require any additional purification steps, resulting in cost-, time-, and material-effectiveness. Kretzer et al. also explored the comparative efficiency of PLGA and Ace-DEX NPs encapsulated with BRP-201 (an effective FLAP (5-Lipoxygenase-activating protein) antagonist) synthesized via the nanoprecipitation method for suppressing leukotriene formation (proinflammatory lipid intermediaries produced via 5-lipoxygenase) in white blood cells and whole blood [19]. PLGA and Ace-DEX NPs both displayed substantially higher efficiency in inhibiting leukotriene formation in neutrophils as compared to the free drug. It was discovered that BRP-2010-loaded Ace-DEX NPs were more promising than PLGA-NPs because of homogeneous formulation formation in the case of Ace-DEX NPs and formation of drug precipitation in PLGA NPs. Moreover, whole blood experiments revealed an advantage for Ace-DEX NPs as encapsulation within Ace-DEX significantly enhanced the potency of BRP-201, particularly during extended incubation periods (greater than or equal to 5 h) and when the blood was incubated with lipopolysaccharide (LPS), a potent inflammatory agent. These findings indicate that Ace-DEX NPs offer a promising strategy for delivering BRP-201 and potentially other therapeutic agents, with improved efficacy and potentially lowered side effects [19].

**Figure 5:**
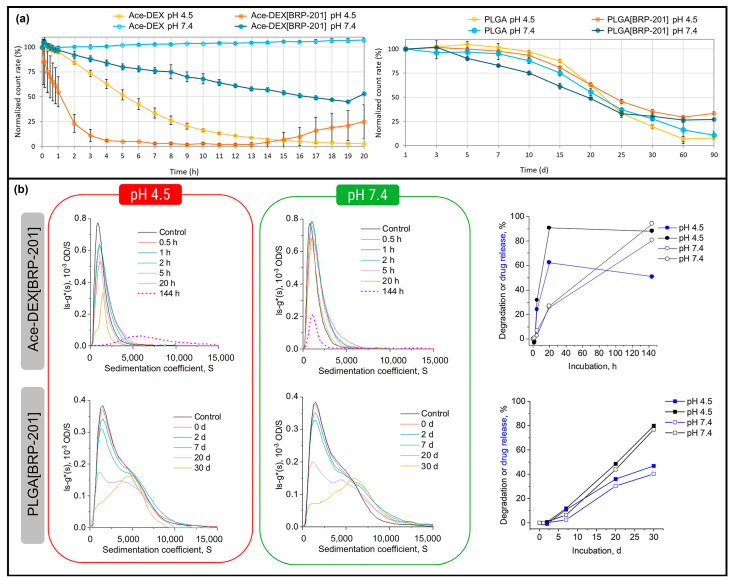
(**a**) Comparison of degradation rate of Ace-DEX NPs and PLGA NPs at pH 4.5 and 7.4, (**b**) Comparison of drug release capability of Ace-DEX NPs and PLGA NPs at pH 4.5 and 7.4. Reproduced from ref. [19], published by Wiley, under the Creative Commons Attribution-CC by 4.0 Attribution 4.0 International Deed.

These comparative studies also reflect that Ace-DEX has the potential to compete and beat the commercially used PLGA in its encapsulation properties [18]. Kretzer et al. established that Ace-DEX particles had a higher drug encapsulation capability as compared to PLGA particles due to its versatility [19]. Encapsulation efficiency (EE) for all PLGA and Ace-DEX NPs with 3% (w w^−1^) BRP-201 drug was >70%. With polymer optimization and 10% (w w^−1^) BRP-201 drug, Ace-DEX particles were able to reach 100% EE. The degradation rate of Ace-DEX and PLGA particles at pH 4.5 and pH 7.4 revealed that half of the Ace-DEX NP degraded within 50 min and 20 h at pH 4.5 and 7.4, respectively, as compared to 20 days and 25 days for pH 4.5 and 7.4, respectively, for PLGA NPs (Figure 5a). The 60% encapsulated drug was released at pH 4.5 as compared to 25% released at pH 7.4 after 20 h in the case of Ace-DEX NPs. However, only 30% encapsulated drug was released at both pH after 20 days in the case of PLGA-NPs (Figure 5b).

### 2.4. HIV-Related Illnesses Treatment

One promising approach involves a novel injectable platform with a high aspect ratio, small ribbon- or tape-like scaffolds known as microconfetti (MC). These MC are engineered by mechanical fragmentation of electrospun nanofibers. This technique offers a unique advantage: the MC’s ribbon- or tape-like particles maintain the capability of electrospun nanofibers to provide persistent drug release. Collier et al. developed a novel injectable release system composed of Ace-DEX microconfetti (Ace-DEX-MC) for the persistent delivery of saquinavir (SQV), an antiretroviral protease inhibitor, used as a treatment for acquired immunodeficiency syndrome (AIDS) and human immunodeficiency virus (HIV) infection [20]. The Ace-DEX-MC system offers both a high drug loading capacity and tunable degradation kinetics, enabling controlled release of SQV. To fabricate the MCs, Ace-DEX, PLGA, and PCL scaffolds were all processed through a high-speed cryomilling technique. Ace-DEX formed well-defined MCs, demonstrating its suitability for the injectable release system. In contrast, the PLGA and PCL polymers fused to form large aggregates, suggesting their incompatibility in forming an efficient injectable release system [20]. Ace-DEX offers unparalleled control over drug release kinetics compared to commonly used biodegradable polymers like PLGA or PCL. This fine-tuning capability is achieved by strategically modifying two key parameters: the hydrolytic stability of the Ace-DEX polymer and the drug loading. This unique flexibility allows for the precise tailoring of release profiles to meet specific therapeutic needs, which is not readily achievable with PLGA or PCL.

## 3. Vaccines

A safe, effective, and efficient vaccine development relies heavily on suitable delivery systems. In this regard, Ace-DEX scaffolds have emerged as a propitious platform due to its inherent biocompatibility and tunable biodegradability. These properties ensure minimal toxicity and allow for the natural breakdown of the carrier material within the body after delivering the vaccine payload. These characteristics eliminate the need for the body to eliminate a foreign substance alongside the vaccine, potentially improving overall vaccine efficacy. Additionally, Ace-DEX NPs can be engineered to target specific immune cells [21,38], further enhancing the immune response triggered by the vaccine. The glass transition temperature (Tg) of Ace-DEX is reported to be approximately 160–190 °C, whereas PLGA and PCL are around 50 °C [39,40,41].

### 3.1. Influenza Vaccines

Ace-DEX scaffolds show tunable release kinetics and steadiness outside the cold-chain. They present a very promising platform for encapsulating proteins or other macromolecules as they maintain encapsulated protein bioactivity even at elevated temperatures as compared to other biopolymers like PCL or PLGA [39,40,41]. This eliminates the need for expensive and logistically challenging cold-chain transportation, making Ace-DEX-based therapies more accessible and cost-effective [39]. For example, Peine et al. documented the CpG (cytosine–phosphate–guanine) encapsulation within Ace-DEX MPs using an emulsion-based method with an encapsulation efficiency of 36.6% [38]. Recently, Batty et al. utilized the electrospray method to encapsulate CpG in the Ace-DEX MPs as an influenza vaccine adjuvant. These CpG-encapsulated Ace-DEX MPs were more potent inducers of proinflammatory cytokine expression (TNF-α, IL-6, and nitric oxide) compared to soluble CpG in the DC2.4 (immortalized murine dendritic cells) and bone-marrow-derived dendritic cells (DCs) [22]. Junkins et al. prepared cyclic guanosine monophosphate-adenosine monophosphate (cGAMP)- (a stimulator of interferon genes (STING) agonist) loaded Ace-DEX MPs using the electrospray method as a vaccine against lethal influenza infection. In vitro experiments demonstrated a 1000-fold increase in type-I interferon responses compared to unencapsulated cGAMP [23]. This potent activation translated to significant in vivo effects when administered intramuscularly, with a 50-fold increase observed. The vaccine also promoted a Th1-associated immune response, known to be crucial for fighting off viral infections. Additionally, the vaccine stimulated the expansion of memory T cells and B cells, necessary for long-term immunity. The effectiveness of the vaccine was further demonstrated by its ability to confer immunity against a lethal influenza virus even months after immunization [23]. Matrix protein 2 (M2e), in conjunction with cGAMP-loaded Ace-DEX microparticles (MPs) as an adjuvant, has been investigated for the development of a potential universal influenza vaccine. [24]. Ace-DEX MP containing both M2e and cGAMP provoked robust humoral (primarily through T-cell mediated responses) and cellular immune responses in vivo when administered via intramuscular injection. These responses translated into a significant defense counter to a lethal influenza virus challenge, indicating promising vaccine efficacy. To enhance the capacity of Ace-DEX MPs to trigger B cell responses against the M2e influenza epitope, Batty et al. chemically conjugated the M2e peptide directly to the surface of the particles [42]. This approach aimed to improve the overall immunogenicity of the M2e vaccine delivery system. These surface-decorated antigen scaffolds could offer protection against a lethal influenza virus challenge with one dose, as compared to the encapsulated M2e, when co-administered with cGAMP-Ace-DEX MPs.

**Figure 6:**
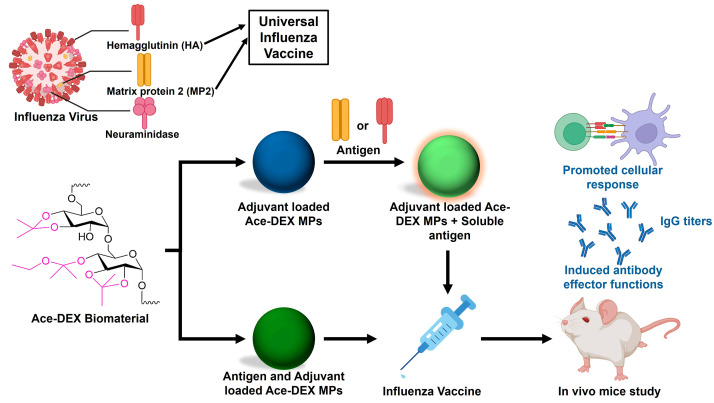
A schematic representation of methods to develop a universal influenza vaccine from Ace-DEX MPs. Modified images from refs. [42,49] with permission from American Chemical Society, Copyright © 2023.

A novel approach to influenza vaccination can be employed using computationally optimized broadly reactive antigen (COBRA) technology. This method designs hemagglutinin (HA) antigens that target conserved regions of the influenza virus, potentially offering broader protection against various strains [43,44,45]. In a recent study, a vaccine using multivalent and monovalent COBRA HA was prepared with cGAMP-encapsulated Ace-DEX MPs which was administered intranasally during in vivo studies. The protection against influenza was evidenced by a decrease in pulmonary viral titers, indicating a reduction in the amount of replicating virus within the lungs in a mouse model. These findings suggest that COBRA vaccines co-delivered with cGAMP MPs may be a promising strategy for developing more effective influenza vaccines [46]. COBRA HA was also conjugated to the exterior of the Ace-DEX MPs via a vinyl sulfone terminal group present on a co-polymer. These COBRA-conjugated Ace-DEX MPs were then combined with cGAMP-encapsulated MPs and administered intramuscularly [47]. The combined delivery system offers the potential for a single-shot, broadly acting influenza vaccine. This combined delivery system induced functional antibodies in the mouse model. These antibodies specifically bind to the virus, preventing its ability to infect healthy cells. Overall, two primary strategies were explored for utilizing Ace-DEX MPs to develop influenza vaccines (Figure 6). The first approach involved co-administration, where separately formulated adjuvant-loaded Ace-DEX MPs were used with the soluble antigen [23,48,49] This method allowed for independent control over the release profiles of each component. Alternatively, encapsulation of the antigen and adjuvant together within the same Ace-DEX MPs was employed [30]. This strategy aimed to achieve a more localized and synchronized delivery of these immunostimulatory molecules, potentially leading to a more potent immune response.

### 3.2. Vaccine for Vector-Borne Diseases

Besides influenza vaccines, Ace-DEX scaffolds have been explored as a vaccine platform in other disease conditions such as the West Nile Virus (WNV) vaccine, the malaria vaccine, SARS-CoV-2 vaccine, and many more [25,26,27,28,50,51]. A promising approach to West Nile Virus (WNV) vaccination was explored by utilizing mast cell activation using Ace-DEX scaffolds. This strategy involved a small molecule activator, MCA ST101036, encapsulated within Ace-DEX MPs [25]. These loaded Ace-DEX MPs were merged with the West Nile Virus envelope III protein (EDIII) and administered subcutaneously. These MPs were able to activate mouse mast cells in vitro, which was confirmed through two key observations: degranulation, which is the release of pre-stored inflammatory mediators by MCs, and the production of proinflammatory cytokines, signaling molecules that support orchestrating the immune response. Vaccination with these MPs induced a balanced Th1/Th2 immune response in vivo. This is evidenced by the production of elevated levels of both IgG1 and IgG2a antibodies. IgG1 antibodies are associated with a humoral immune response, effective against extracellular pathogens. IgG2a antibodies, on the other hand, play a crucial role in activating macrophages and cytotoxic T cells, enhancing cellular immunity. This approach represents a novel avenue for WNV vaccine development. Stiepel et al. demonstrated the synthesis of a potential malaria vaccination by adsorbing the Merozoite surface protein 2 (MSP2) antigen onto Ace-DEX MPs [26]. MSP2-absorbed Ace-DEX MPs were able to stimulate a Th1-biased response to fighting an intracellular pathogen, Plasmodium falciparum, in vivo via an intramuscular (IM) injection and demonstrated a promising platform for a malaria vaccine.

### 3.3. Tolerogenic Vaccines

Rapamycin-loaded Ace-DEX particles have emerged as a promising platform for tolerogenic vaccines. These versatile systems offer a unique advantage: the core delivery system remains constant, while the disease-specific autoantigen can be modified easily. This adaptability allows for targeted development of tolerogenic vaccines against various autoimmune diseases. Chen et al. engineered rapamycin- and ovalbumin-encapsulated Ace-DEX MPs by double emulsion, which showed a distinct anti-inflammatory response in vitro and a protective effect against multiple sclerosis (encephalomyelitis) in a murine model during in vivo studies [27]. In vivo studies, exploring type 1 diabetes prevention, demonstrate the effectiveness of this platform. When loaded with the relevant autoantigen, Ace-DEX particles successfully induced tolerance in mice when administered subcutaneously, indicating the potential of this approach for preventing type 1 diabetes [50]. These findings emphasize the promise of Ace-DEX particles as a versatile platform for developing personalized tolerogenic vaccines against a broad range of autoimmune disorders.

Resiquimod-loaded Ace-DEX MC has been explored as an injectable vaccine platform and as a potential tool for immune stimulation [51]. In vitro studies have shown that Ace-DEX MC effectively stimulates the inflammatory cytokines production in bone-marrow-derived dendritic cells (DCs). This cytokine production is a crucial step in activating the immune response. Furthermore, these studies reveal a significant advantage of the MC delivery system: no additional cytotoxicity was observed in the DCs compared to controls. This suggests that resiquimod-loaded MCs offer a promising approach for stimulating the immune system with minimal negative effects on cellular health.

### 3.4. Universal SARS-CoV-2 Vaccines

**Figure 7:**
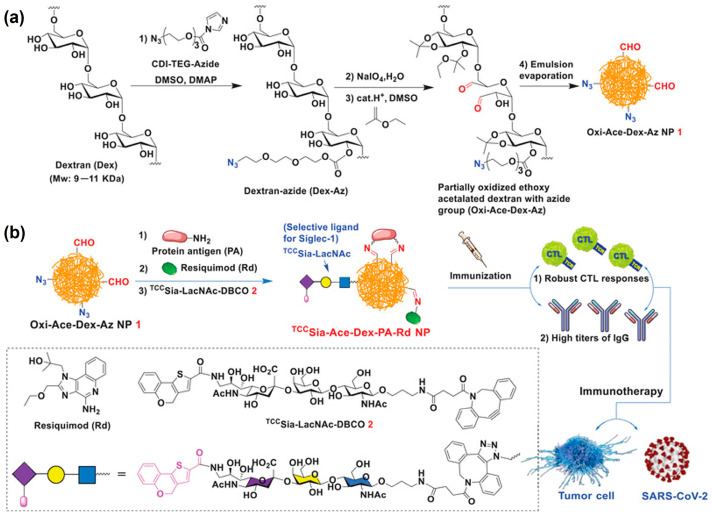
(**a**) Oxi-Ace-DEX-Az nanoparticles (NPs) synthesis scheme. (**b**) Representation diagram showing of chemical conjugation of resiquimod (Rd) and protein antigen (PA) with Oxi-Ace-DEX-Az NPs. Reproduced from ref. [28], published by Wiley, under the Creative Commons Attribution-CC by 4.0 Attribution 4.0 International Deed.

A promising new platform for vaccine development has emerged with the introduction of Ace-DEX glyconanoparticles by Gao et al. (Figure 7) [28]. This innovative approach utilizes partially oxidized, azide-functionalized Ace-DEX nanoparticles. A key feature of this platform is the ability to chemically conjugate targeting ligands to the polymer chain. This targeted delivery system holds promise for enhancing both cytotoxic T lymphocyte (CTL) and IgG antibody responses when administered subcutaneously, potentially leading to more effective vaccines. Along with the anticancer vaccine, this versatile platform has been utilized to make unprecedented and universal SARS-CoV-2 vaccines which show a superior anti-RBD IgG response and robust anti-N CTL response against SARS-CoV-2.

## 4. Cancer Immunotherapy

Cancer immunotherapy involves a novel approach to harnessing the body’s immune system to identify and terminate cancer cells [52,53]. Unlike traditional therapies which target cancer cells directly, immunotherapy empowers the immune system to develop a targeted and long-lasting response against the malignancy [52]. This shift in treatment philosophy has yielded promising results for various cancer types, offering renewed hope for patients. Ace-DEX-MPs demonstrate the ability to enhance the presentation of major histocompatibility complex class I (MHC-I) molecules on antigen-presenting cells (APCs) [54].

**Figure 8:**
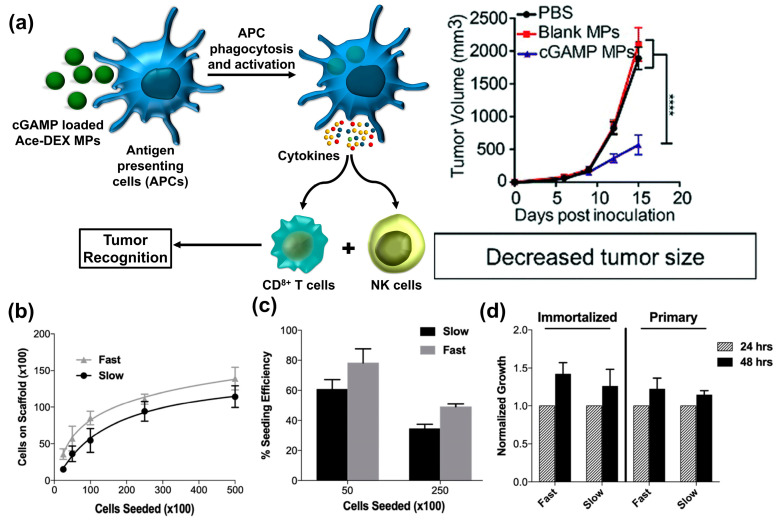
(**a**) Schematic presentation of cGAMP-Ace-DEX MPS action after administration in vivo, and B16.F10 melanoma tumor volume after treatment with cGAMP-Ace-DEX MPs. (**** *p* < 0.0001). Reproduced from ref. [54], with permission from Elsevier, Copyright © 2019. (**b**) Cell-carrying capability of Ace-DEX gelatin nanofibers with C17.2 neural stem cell (NSCs). at 24 h (**c**) Efficacy in cell seeding. (**d**) Cell growth on Ace-DEX gelatin nanofibers at 48 h. Reproduced from ref. [10], with permission from Elsevier, Copyright © 2020.

MHC-I plays a crucial role in priming cytotoxic T lymphocytes (CTLs), which are a key element of the immune response against cancer cells. Watkins-Schulz et al. reported the use of cGAMP-encapsulated Ace-DEX MPs for cancer immunotherapy [54]. These micron-sized MPs (ranging from 0.5 to 2 μm) demonstrated a passive targeting towards antigen-presenting cells (APCs). In vivo studies using the B16F10 melanoma model (a fast-growing model) revealed that the observed antitumor efficiency was primarily due to natural killer (NK) cells. Interestingly, the antitumor response in the TNBC model (a slower-growing model) exhibited dependence on NK cells and CD8+ T cells together, suggesting a potential influence of tumor growth rate on the immune cell response activated by cGAMP-loaded Ace-DEX MPs (Figure 8a) [54]. Simpson et al. incorporated phosphatidylserine (PS) into Ace-DEX MPs and assessed their interaction with peritoneal macrophages (PMacs) [21]. PS’s ability to promote cell tolerance, including the anti-inflammatory cytokine IL-10 production, and the expression of genes associated with the M2 macrophage phenotype were examined with PS-Ace-DEX MPs. Along with this, 2-(1′H-indole-3′-carbonyl)-thiazole-4-carboxylic acid methyl ester (ITE), an activator of the aryl hydrocarbon receptor (AhR), was integrated into Ace-DEX MPs to evaluate the M2 and IL-10 genes expression in PMacs. Furthermore, the combined PS/ITE-Ace-DEX MPs were assessed for their ability to suppress T cell priming and Th1 polarization, a key step in inflammatory responses. The study’s findings suggested that PS/ITE-MPs effectively stimulate the expression of anti-inflammatory cytokines and suppress inflammation in LPS-activated PMacs. Additionally, PS/ITE-MPs generated the expression of the anti-inflammatory enzyme IDO1 along with suppression of macrophage-mediated T cell priming and Th1 polarization. These results indicate that PS- and ITE-loaded Ace-DEX MPs hold promise as a therapeutic tool for managing inflammatory conditions.

## 5. Tissue Engineering

One of the most important criteria in tissue engineering is that the rate of degradation of scaffold should meet the rate of formation of tissue to be incorporated effectively into the surrounding tissue where they have been used [55]. Scaffold degradation has been regulated previously in many biomaterials (natural and synthetic) by exploring different blending ratios of polymers [56], crosslinking using chemical conjugations [57], different fabrication parameters [58], and different solvents during fabrication processes [59]. In case of Ace-DEX biomaterials, the degree of acetylation substantially affects its hydrophilicity, biocompatibility, and degradation rate. Higher levels of acetylation lead to enhanced hydrophobicity, mechanical properties, and high degradation rate. Higher-molecular-weight Ace-DEX biomaterials have better mechanical strength but slow degradation rate, which can potentially affect cell–material interactions. However, by fine-tuning these factors, the Ace-DEX can be easily modified into the scaffolds which can mimic the natural extracellular matrix (ECM) and provide a supportive microenvironment that facilitates cellular growth, differentiation, and successful integration with surrounding tissues [10]. These properties make them highly promising candidates for applications in regenerative medicine and tissue engineering. A particularly exciting area of exploration is their potential use in neural stem cell (NSC) therapy for glioblastoma (GBM). Moore et al. demonstrated that Ace-DEX blended with gelatin and electrospun into nanofibers significantly improved the efficacy of NSC implantation and promoted its long-term persistence within the tumor cavity as compared to NSCs’ direct injection (DI) in suspension (Figure 8b–d) [10]. Nanofibers with both slow and fast degradation rates significantly enhanced NSC implantation efficiency, with increases of 3.03-fold and 2.87-fold, respectively, compared to DI. Furthermore, both scaffold types demonstrated significantly improved persistence and long-term survival (up to 120 days) of NSCs compared to the DI approach. These findings suggest that Ace-DEX scaffolds hold great promise for improving the effectiveness of NSC therapy for GBM.

## 6. Current Limitations

Ethoxy acetalated dextran or Ace-DEX is a versatile polymer with a broad range of potential biomedical applications. However, challenges and limitations still remain in the development and synthesis of this biomaterial which currently limits its use. The standardization of polymer synthesis and characterization is a first important step to minimize batch variations, which is still not readily available. As mentioned earlier, Ace-DEX’s degradation rate can be modified by changing the degree of acetylation. However, achieving precise control over its degradation kinetics is still a challenge which can impact the release of encapsulated drugs or biomolecules. Due to the relatively recent introduction of Ace-DEX biomaterials as compared to conventionally used polymers, the optimization of fabrication parameters, characterization of degradation products, thorough biocompatibility and toxicity assessment, scale-up, and production reproducibility are all vital areas for further investigation. Ace-DEX may trigger an immune response, especially when used for the treatment of chronic diseases where prolonged drug administration is required or disease conditions where high drug concentrations are needed as part of treatment. Potential unintended acetal hydrolysis during synthesis, scaffold fabrication, or storage is an important aspect which needs to be addressed. While recent preclinical studies have demonstrated the potential of Ace-DEX scaffolds, clinical experience is still limited, and careful evaluation of short-term and long-term toxicity is necessary before clinical use. These improvements will ultimately enhance the efficacy and reliability of Ace-DEX scaffolds.

## 7. Conclusions and Future Perspective

In conclusion, Ace-DEX has emerged as a versatile and promising biomaterial for various therapeutic applications, such as drug delivery, immunotherapy, tissue engineering, and vaccines. Its facile synthesis and capacity for chemical modification allow for tailoring its physicochemical and biological properties to specific needs. Additionally, Ace-DEX can be fabricated into diverse forms and sizes, catering to different delivery routes, targets, and applications. Compared to commercially available polymeric drug carriers like PLGA, Ace-DEX offers several advantages for clinical translation. Firstly, its similar solubility profile allows for fabrication using established procedures, streamlining the development process. Secondly, Ace-DEX displays a unique combination of properties, tunable pH-dependent drug release, biodegradability, and biocompatibility, features which are highly attractive for future clinical use. Despite the current challenges, Ace-DEX polymers hold promise as biomaterials for therapeutic applications. However, compared to established options, Ace-DEX research is still in its early stages. Ace-DEX biomaterials have the potential to revolutionize tissue engineering (neural, skin, and other soft tissues) and the field of regenerative medicine. While some studies have explored how to chemically modify Ace-DEX for use as a targeted drug delivery platform, this exciting area presents vast opportunities for further investigation to explore potentially new therapeutic applications. Continued research is the key to unlocking the full potential of Ace-DEX as a versatile and valuable biomaterial in the future.

## Figures and Tables

**Table 1:** Selective summary of loaded drug, scaffold types, fabrication methods, and therapeutic applications of Ace-DEX biomaterial.

Drug/Load	Ace-DEX Carrier	Fabrication Method	Application	Ref.
Gelatin	Nanofiber	Electrospinning	Tissue engineering	[10]
Doxorubicin	Nanofiber	Electrospinning	Glioblastoma interstitial therapy	[11]
AR-12	Microparticles	Emulsion solvent evaporation	Treatment for visceral leishmaniasis	[12]
AR-12	Microparticles	Emulsion solvent evaporation	Treatment for Salmonella infection	[13]
AR-12 and gentamicin	Microparticles	Emulsion solvent evaporation	Treatment for Francisella infection (rabbit fever)	[14]
Collagenase	Nanoparticles	Emulsion solvent evaporation	Cancer treatment	[15]
Paclitaxel	Nanofiber	Electrospinning	Glioblastoma interstitial therapy	[16]
Paclitaxel and Everolimus	Nanofiber	Electrospinning	Glioblastoma interstitial therapy	[17]
BRP-187	Nanoparticles	Emulsion solvent evaporation, microfluidics and nanoprecipitation	Anti-inflammatory	[18]
BRP-201	Nanoparticles	Nanoprecipitation	Inhibition of leukotriene formation	[19]
Saquinavir	Microconfetti	Electrospinning	HIV treatment	[20]
Phosphatidylserine (PS)	Microparticles	Emulsion solvent evaporation	Cancer immunotherapy	[21]
Cytosine–phosphate–guanine (CpG)	Microparticles	Electrospray	Influenza vaccine	[22]
Cyclic guanosine monophosphate-adenosine monophosphate cGAMP	Microparticles	Electrospray	Influenza vaccine	[23]
Matrix protein 2 (M2e) and cGAMP	Microparticles	Emulsion solvent evaporation	Influenza vaccine	[24]
MCA ST101036, R529877, ST048871, and ST027688	Microparticles	Nanoprecipitation	West Nile Virus (WNV) vaccine	[25]
Merozoite surface protein 2 (MSP2)	Microparticles	Electrospray	Malaria Vaccine	[26]
Rapamycin and Ovalbumin	Microparticles	Double emulsion method	Multiple sclerosis (encephalomyelitis) vaccine	[27]
Resiquimod	Microparticles	Emulsion and evaporation	SARS-CoV-2 vaccines	[28]
